# Impairments of Thalamic Nuclei in Idiopathic Generalized Epilepsy Revealed by a Study Combining Morphological and Functional Connectivity MRI

**DOI:** 10.1371/journal.pone.0039701

**Published:** 2012-07-11

**Authors:** Zhengge Wang, Zhiqiang Zhang, Qing Jiao, Wei Liao, Guanghui Chen, Kangjian Sun, Lianfang Shen, Maoxue Wang, Kai Li, Yijun Liu, Guangming Lu

**Affiliations:** 1 Department of Medical Imaging, Jinling Hospital, Nanjing University School of Medicine, Nanjing, China; 2 Department of Neurology, Jinling Hospital, Nanjing University School of Medicine, Nanjing, China; 3 Department of Neurosurgery, Jinling Hospital, Nanjing University School of Medicine, Nanjing, China; 4 Center for Cognition and Brain Disorders and the Affiliated Hospital, Hangzhou Normal University, Hangzhou, China; 5 Department of Pharmacology, Suzhou University, Suzhou, China; 6 Department of Psychiatry and McKnight Brain Institute, University of Florida, Gainesville, Florida, United States of America; Hangzhou Normal University, China

## Abstract

**Objective:**

Neuroimaging evidence suggested that the thalamic nuclei may play different roles in the progress of idiopathic generalized epilepsy (IGE). This study aimed to demonstrate the alterations in morphometry and functional connectivity in the thalamic nuclei in IGE.

**Methods:**

Fifty-two patients with IGE characterized by generalized tonic-clonic seizures and 67 healthy controls were involved in the study. The three-dimensional high-resolution T1-weighted MRI data were acquired for voxel-based morphometry (VBM) analysis, and resting-state blood-oxygenation level functional MRI data were acquired for functional connectivity analysis. The thalamic nuclei of bilateral medial dorsal nucleus (MDN) and pulvinar, as detected with decreased gray matter volumes in patients with IGE through VBM analysis, were selected as seed regions for functional connectivity analysis.

**Results:**

Different alteration patterns were found in functional connectivity of the thalamic nuclei with decreased gray matter volumes in IGE. Seeding at the MDN, decreased connectivity in the bilateral orbital frontal cortex, caudate nucleus, putamen and amygdala were found in the patients (P<0.05 with correction). However, seeding at the pulvinar, no significant alteration of functional connectivity was found in the patients (P<0.05 with correction).

**Conclusions:**

Some specific impairment of thalamic nuclei in IGE was identified using morphological and functional connectivity MRI approaches. These findings may strongly support the different involvement of the thalamocortical networks in IGE.

## Introduction

Generalized tonic-clonic seizures (GTCS) is the most frequent subtype of idiopathic generalized epilepsy (IGE), which is characterized by rigid stiffening of the limbs, followed by violent bilateral spasms, and loss of consciousness. Patients with IGE typically present bilaterally generalized synchronous discharges in electroencephalography (EEG) and without apparent abnormalities in routine MRI examinations [1]. It is currently proposed that the thalamocortical circuits play an important role in the neuropathophysiological mechanism of IGE [2]. Neuroimaging studies have demonstrated localized abnormalities in the thalamus and cortical structures. Morphological studies have demonstrated gray matter deficits in the thalamus and cortical structures [3], [4]. EEG combined functional MRI (EEG-fMRI) studies have also shown thalamic activation along with cortical deactivation responding to generalized epileptic discharges [5]–[7]. Moreover, brain connectivity studies indicate the thalamocortical circuit abnormalities in IGE from perspective of brain network. Animal studies demonstrated that spike-wave discharges may originate in the neocortex and lead to synchronization of thalamocortical loops [8]. A MRI study using structural covariance network found that the gray matter volume of the thalamus is correlated with the cortical thickness of frontal, limbic, and occipital regions [9]. Resting-state fMRI studies with functional connectivity analysis have shown wide network impairments and reorganization in IGE [10], [11]. A recent work of our group found disrupted topological organization in large-scale brain functional and structural networks with hub of thalamus in IGE [12].

Moreover, it has been proposed that different thalamic nuclei may play specific roles in the pathophysiological progression of IGE [13]. Tyvaert and colleagues found that the activity of centromedian and parafascicular nucleus was earlier than the anterior nucleus during generalized spike wave discharges. They considered that the centromedian and parafascicular nucleus might be involved in epileptic discharge initiation or early propagation, while the anterior nuclei only play a role in its maintenance [14]. The medial pulvinar nucleus has also been found to participate in seizures propagation [15]. Given the evidence that the thalamic nuclei have different imaging demonstrations, it would be interesting to know whether there are different affections of the brain networks associated with the thalamic nuclei in IGE.

In this study, we firstly detected the affected thalamic nuclei in the patients with IGE-GTCS by using voxel-based morphometry (VBM) analysis on structural MRI data, and secondly, investigated the altered brain networks of these thalamic nuclei through functional connectivity analysis on resting-state blood oxygenation level-dependent functional MRI data. This study is designed to determine the patterns of functional and structural impairments of brain networks associated with different thalamic nuclei in IGE-GTCS.

## Materials and Methods

### Subjects

Fifty-two patients with Idiopathic generalized epilepsy characterized by generalized tonic clonic seizures (IGE-GTCS) were included in this study. Their demographic and clinical information were summarized in the table 1. All patients were diagnosed as IGE with only GTCS according to the criteria of International League Against Epilepsy (ILAE) classification: (1) with typical clinical symptoms of generalized tonic-clonic seizures, including tonic extension of the limbs, followed by a clonic phase of rhythmic jerking of the extremities, loss of consciousness during seizures; (2) presence of generalized polyspike-wave in their interictal scalp EEG; and (3) no focal abnormality in the structural MRI. Thirty-seven out of the 52 patients were treated with anti-epileptic drugs (AEDs) (monotherapy/polytherapy, 26/11; valproic acid, 21; phenytoin, 12; topiramate, 6; Carbamazepine, 10). Moreover, 67 healthy subjects were recruited as controls. None of them had history of neurological or psychiatric disorder. Written informed consent was obtained from all participants. The study was approved by the local medical ethics committee at Jinling Hospital, Nanjing University School of Medicine.

**Table 1:** Characteristics of the IGE-GTCS patients and the healthy controls.

Characteristics	IGE-GTCS(n = 52)	Healthy Controls (n = 67)	p
\	Mean ± SD	Mean ± SD	\
Gender (male/female)	31/21	39/28	0.87^+^
Age (year)	24.96±7.28	23.13±2.28	0.09^++^
Handedness (right/left)	52/0	67/0	\
Duration (year)	7.32±7.53	\	\

p^+^was obtained by Pearson Chi-Square,

p^+^was obtained by two-sample t test.

**Figure 1:**
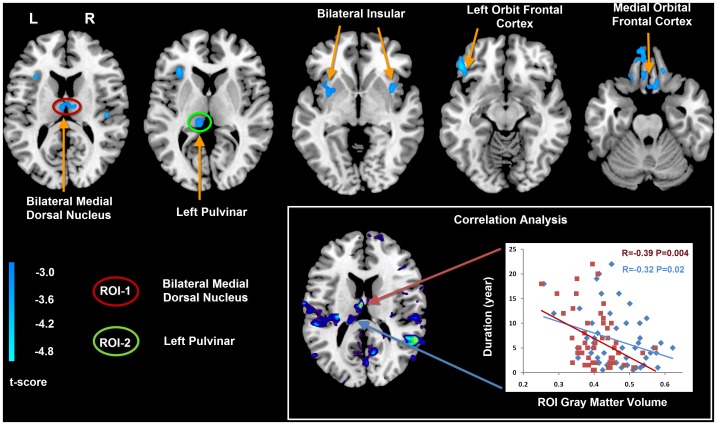
Gray matter difference between IGE-GTCS and healthy controls, and correlation analysis between gray matter volume and epilepsy duration. The cold color denotes the brain regions having reduced gray matter volume in IGE-GTCS patients. Maps threshold were set at *P*<0.01, with AlphaSim correction. According to this VBM comparison results, the bilateral medial dorsal nuclei (Red circle) and the left pulvinar (Green circle) were defined as ROIs for the subsequent functional connectivity analyses. In addition, the left medial dorsal nuclei and pulvinar showed negative correlation between gray matter volume and epilepsy duration. The red block represent the gray matter volume of IGE-GTCS patients in left medial dorsal nucleus (R = −0.39, P = 0.004), the blue rhomboid represent the gray matter volume of IGE-GTCS patients in left pulvinar (R = −0.32, P = 0.02).

### MRI Data Acquisition and Analysis

#### Data acquisition

All structural and functional MRI data of the patients and controls were collected using a Siemens 3T Trio scanner (Siemens Medical Systems, Erlangen, Germany) with an eight-channel phased array head coil in Jinling Hospital. High-resolution T1-weighted structure data were acquired using a Three Dimensional Magnetization Prepared Rapid Acquisition Gradient-echo (3D MPRAGE) sequence: TR/TE = 2300 ms/2.98 ms, FA = 9°, matrix = 256×256, FOV = 256×256 mm^2^, and slice thickness = 1 mm. The matrix size was automatically interpolated in-plane to 512×512, and the final resolution was 0.5×0.5×1 mm^3^. Resting-state fMRI data were acquired using Gradient Echo type Echo Planar Imaging (GRE-EPI) sequence: TR/TE = 2000 ms/30 ms, FA = 90°, matrix = 64×64, FOV = 24×24 cm^2^, and slice thickness = 4 mm, slice gap = 0.4 mm. A total of 30 slices were used to cover the whole brain. Each section contained 250 volumes. All patients and healthy subjects were instructed to relax, hold still, and keep their eyes closed during the resting-state functional MRI examination. All patients were absent of seizures symptoms, and were in interictal state.

### Voxel-Based Morphometry Analysis

The 3D T1-weighted images were analyzed with VBM-DARTEL, using SPM8 software package (http://www.fil.ion.ucl.ac.uk/spm) implemented in MATLAB 2006b (Math Works, Natick, MA, USA). The images were segmented into gray matter, white matter and cerebrospinal fluid using the unified standard segmentation option in SPM8. After segmentation, the gray matter images were normalized using the DARTEL toolbox following John Ashburner’s chapter in the standard version [16]. Spatially normalized images were then smoothed with 4 mm FWHM (full width at half maximum) Gaussian kernel.

**Figure 2:**
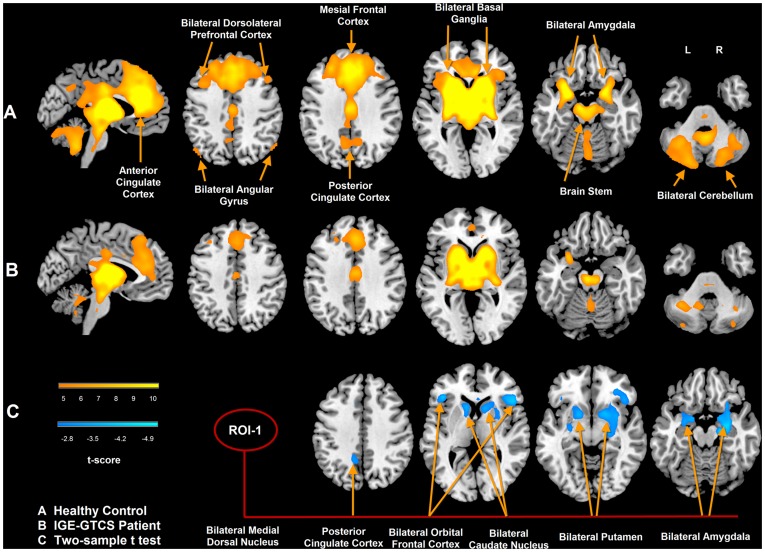
Functional connectivity of medial dorsal nucleus in IGE-GTCS patients and healthy subjects. A: functional connectivity map of MDN in healthy controls; B: functional connectivity map of MDN in patients with IGE-GTCS; C: group difference of functional connectivity map of MDN. The warm color represents that the brain regions showed positive correlation with bilateral medial dorsal nucleus (p<0.05, FWE correction). The cool color represents that the brain regions showed decreased functional connectivity in IGE-GTCS patients, the threshold was set at p<0.05 after AlphaSim correction.

Statistical analysis of the data was conducted in two steps. First, for each subject, gray matter, white matter and cerebrospinal fluid absolute volumes were calculated by estimating these segments. The total intracranial volume was obtained by summing the gray matter, white matter and cerebrospinal fluid volumes. Second, voxel-by-voxel based comparisons of gray matter volume were performed between the IGE-GTCS and healthy groups using two-sample *t*-tests. The significance levels were set at p<0.01 with AlphaSim correction (combined height threshold p<0.001 and a minimum cluster size = 54 in gray matter template) in the REST software (http://www.restfmri.net/forum/REST V1.7) [17]. Age, gray matter volume and total intracranial volume were regressed out as nuisance covariates. The thalamic nuclei with significant reduced gray matter volume were extracted as regions of interest (ROIs) for functional connectivity analysis.

**Figure 3:**
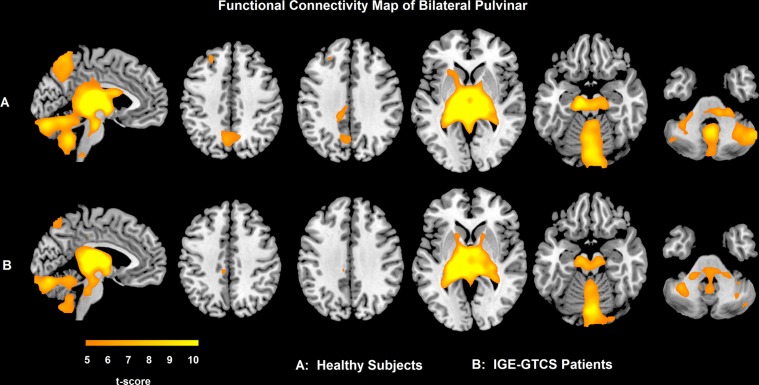
Functional connectivity of pulvinar in IGE-GTCS patients and healthy subjects. A: functional connectivity map of pulvinar in healthy controls; B: functional connectivity map of pulvinar in patients with IGE-GTCS; The warm color denotes that the brain regions showed positive correlation with bilateral pulvinar (p<0.05, FWE correction).

Voxel-based correlation analysis between gray matter volume and duration of epilepsy in patients was performed to estimate the impairing effect of epilepsy on the thalamus. The results were corrected at p<0.05 using AlphaSim correction (combined height threshold p<0.01 and a minimum cluster size = 65 in gray matter template). Individual age, gray matter volume and total intracranial volume were regressed out as confounding covariates.

### Functional Connectivity Analysis

The resting-state fMRI data were preprocessed using SPM8 software package (http://www.fil.ion.ucl.ac.uk/spm) implemented in MATLAB 2006b (Math Works, Natick, MA, USA) platform. The first 10 volumes of each participant were discarded. The remaining 240 scans were slice-time corrected and then realigned to the first volume to correct head motions. The data whose motion exceeded 1.5 mm or rotation exceeded 1° during scanning were excluded. All realigned images were spatially normalized to the MNI template and resampled to the voxel size of 3×3×3 mm^3^. At last, the data were smoothed with an isotropic 8 mm FWHM Gaussian kernel.

Functional connectivity was examined using a ROI based method. The thalamic nuclei with significantly reduced gray matter volume were extracted as regions of interest (ROIs: bilateral medial dorsal nucleus and bilateral pulvinar) for functional connectivity analysis (Fig. 1). These ROIs were then resliced to the MNI coordinate with resting-state data. The averaged time course was obtained from the ROI and the correlation analysis was performed in a voxel-wise way to generate the functional connectivity map. The systematic drift of the baseline signal was removed by computing the least-squares fit of a straight line to each voxel’s time series and subtracting the resulting function from the time series, and then the data were temporally bandpass filtered (0.01∼0.08 Hz) to reduce the effect of low and high frequency physiological noise. In addition, the global mean signal, six motion parameters, the cerebrospinal fluid, and the white matter signals were removed as nuisance variables to reduce the effects of head motion and non-neuronal BOLD fluctuations. The correlation coefficient map was converted into z map by Fisher’s r-to-z transformation to improve the normality. Using one-sample *t*-test, we found the distribution map of functional connectivity of these ROIs (p<0.05, FWE correction) for both controls and patients. Then, two-sample *t*-tests were used to find the differences between-group, the multiple comparison corrections were performed at p<0.05 using AlphaSim (combined height threshold p<0.01 and a minimum cluster size = 65 in gray matter template).

## Results

### Morphometry Analysis

Compared with the healthy controls, the patients with IGE-GTCS showed significantly decreased gray matter volume in the bilateral thalamus (specifically in the medial dorsal nucleus and pulvinar), orbit frontal cortex, insular and cerebellum (Fig. 1). Moreover, both the medial dorsal thalamic nucleus and pulvinar showed negative correlation between gray matter volume and epilepsy duration (Fig. 1). No increased gray matter volume was found in the patients with IGE-GTCS.

### Functional Connectivity Analysis

According to the VBM comparison results, the medial dorsal nucleus (MDN) and pulvinar, which showed significant gray matter reduction in the patients, were selected as seed regions for functional connectivity analysis on resting-state fMRI data. Seeding at the MDN, the functional connectivity maps of the patients and controls both showed significant positive correlation in the bilateral cortical structures (dorsolateral prefrontal cortex, medial frontal cortex, and anterior cingulate cortex), basal ganglia and cerebellum (Fig. 2). Compared with the controls, the patients showed decreased functional connectivity in the bilateral caudate nucleus, putamen, amygdala, and orbital frontal gyrus (Fig. 2). No increased functional connectivity was found in patients with IGE-GTCS.

Seeding at the pulvinar, the functional connectivity maps of the patients and controls both showed significant positive correlation in the bilateral precuneus, the brainstem and the cerebellum (Fig. 3). There was no significant difference between healthy control and patient group for the functional connectivity map of pulvinar.

## Discussion

This study combined voxel-based morphometry and functional connectivity analyses to investigate the morphological and functional network alterations of thalamic nuclei in IGE-GTCS. Compared with the healthy controls, the patients showed decreased gray matter volumes in the medial dorsal nucleus (MDN) and pulvinar, indicating that both the MDN and pulvinar have structural impairments in IGE-GTCS. However, in the functional connectivity analysis, the MDN and pulvinar showed distinct alteration patterns in IGE-GTCS. The brain network seeding at MDN showed decreased connectivity in the bilateral caudate nucleus, putamen, amygdala, and orbital frontal gyrus; whereas, the brain network seeding at the pulvinar showed no alteration of functional connectivity in patients with IGE-GTCS. These results may suggest distinguished impairments between the MDN and pulvinar in the brain networks.

Despite there is no structural abnormality in IGE-GTCS upon visual inspection on routine MRI examination, the quantitative MRI technique, i.e., voxel-based morphometry is able to detect some subtle morphological abnormalities. Previous studies of voxel-based morphometry analysis have demonstrated gray matter deficits in the thalamus and cortical structures, which may indicate neuron loss in thalamocortical network in IGE-GTCS [3], [4], [9], [18]. The decreased gray matter volume in the thalamus, frontal cortex, insular and cerebellum as revealed in this study are consistent with the previous findings. Moreover, this study further detected different morphological alterations of the thalamic nuclei in IGE-GTCS, specifically in the MDN and pulvinar nuclei. Previous animal experiment has suggested the MDN is involved in the mechanism of spike and wave complexes and propagation of seizure [19]. Hodaie and colleges have found bursting activity of neurons in MDN in epilepsy patients with generalized or second generalized seizure [20]. Restricted diffusion was found in the pulvinar in patients with generalized seizures [21]. Voxel-wise correlation analysis indicated negative correlation between gray matter volume and epilepsy duration in the left MDN and left pulvinar.

Although the MDN and pulvinar showed structural deficits, whether there are different affections of the brain networks associated with these thalamic nuclei in IGE is unknown. Moeller and colleagues have found increased MDN-cerebellum connectivity, but pulvinar was not selected as the functional connectivity seed since it was not active during the generalized spike wave discharges [22]. Our results showed structural impairments in both MDN and pulvinar, and these nuclei exhibited distinct functional connectivity alteration patterns in IGE-GTCS. The brain network seeding at the MDN showed bilaterally decreased connectivity in the regions of caudate nucleus, putamen, amygdala, and orbital frontal gyrus; whereas, the brain network seeding at the pulvinar showed no significant alteration of functional connectivity in patients with IGE-GTCS. The MDN showed both gray matter deficits and functional connectivity decrease; however the pulvinar showed only gray matter deficits with no identifiable functional abnormalities observed in our study. These different patterns of impairments of MDN and pulvinar may be related with the different functions of MDN and pulvinar.

The structural connectivity and functional connectivity between MDN and frontal cortex has been suggested in recent neuroimaging studies [23]–[25]. The MDN and frontal circuit are involved in cognitive functions, such as memory, executive function, and planning [26], which are also impaired in IGE patients [27]. Consequently, the decreased functional connectivity of MDN and frontal circuit may cause cognition impairment in IGE-GTCS patients. The caudate and putamen are the main nuclei of basal ganglia, which play important roles in modulation of seizures [28], [29]. Luo and colleagues studied the resting state basal ganglia network in IGE patients, and found increased functional connectivity in bilateral caudate and putamen during interictal discharge period [30]. The diffusion tensor imaging study indicated that microstructure changes of caudate nucleus and putamen were related to the chronic epileptic activity [31]. Basal ganglia was also involved in the movement execution function and related to ictal dystonia in epilepsy patients [13], [32]. The reduced functional connectivity in caudate nucleus and putamen may be related to the impairment of ictal tonic-clonic in IGE-GTCS patients. The reduced functional connectivity of MDN may be resulted from the alteration of nodes (caudate nucleus, bilateral putamen and frontal cortex) in MDN associated network. However, the pulvinar was found involved in visual information processing [33]. Recent study showed defective inhibition in the visual system of photosensitive patients with IGE [34], which was rarely reported in IGE with only GTCS.

Network concept features the new version of epilepsy classification, generalized epileptic seizures are conceptualized as originating at some point within, and rapidly engaging, bilaterally distributed networks [35]. Although the source of generalized epilepsy is still unclear, thalamus, basal ganglia, and cortical circuits are involved in the process of generalized epilepsy [6]. Previous studies investigated the cortical or basal ganglia network separately [10], [30]. We found the MDN and MDN-basal ganglia-frontal circuit were impaired in IGE-GTCS. The specific thalamocortical circuit alteration may be crucial in IGE.

Several methodological issues should be considered. Firstly, we did find negative functional connectivity of MDN and pulvinar, which predominately located in the parietal and occipital lobes. Previous studies have suggested that the negative correlations were strongly associated with the global signal removal [36]. Considering the debate about the physiological significance of the negative connectivity [37], [38], we did not show the results of negative connectivity in the present study. Secondly, our results showed the regions with decreased connectivity in MDN were not always identified with structural changes. Although the association between the structural alterations of gray matters and the functional connectivity was supported by increasing number of evidence[39]–[41], on the other hand, studies also reported that there was inconsistency between the structural and functional impairments [42], [43]. We speculated that the decreased functional connectivity of MDN may result from not only the impairment of MDN, but also from the alteration of some nodes (caudate nucleus, bilateral putamen and frontal cortex) in MDN associated network. The relationship between gray matter volume and functional connectivity need to be further studied.

There are several limitations in our study. Firstly, the possible effect of AEDs on brain function is a confounding factor, as heterogeneous of AEDs administration in patients made it difficult to estimate the effects of AEDs on functional connectivity. Secondly, our data were acquired without simultaneous EEG, we could not evaluate the possible effects of interictal epileptic discharges. Thirdly, in the present study, we used a relatively low sampling rate (TR = 2 s) for multiple (30 slices) acquisitions. Under this sampling rate, respiratory and cardiac fluctuations may still be a problem for fMRI time series, despite a band-pass filtering in the range 0.01∼0.08 Hz is used to minimize these influences. The respiratory and cardiac fluctuations may reduce the specificity of low frequency fluctuations to functional connected regions [44]. Finally, the decreased functional connectivity may simply be attributed to the reduced number of neurons in gray matter. However, this speculation is not supported by the finding that the pulvinar exhibited only gray matter deficits but no significant functional impairment observed.

### Conclusion

This study combined voxel-based morphometry and functional connectivity analyses to investigate the morphological and functional network alterations of thalamic nuclei in IGE-GTCS. We demonstrated different impairments of MDN and pulvinar in IGE. These findings may suggest MDN and MDN-basal ganglia-frontal circuit involved in pathogenesis and development of IGE.
